# Virtual Fencing Technology Excludes Beef Cattle from an Environmentally Sensitive Area

**DOI:** 10.3390/ani10061069

**Published:** 2020-06-20

**Authors:** Dana L. M. Campbell, Jackie Ouzman, Damian Mowat, Jim M. Lea, Caroline Lee, Rick S. Llewellyn

**Affiliations:** 1 Agriculture and Food, Commonwealth Scientific and Industrial Research Organisation (CSIRO), Armidale, NSW 2350, Australia; jim.lea@csiro.au (J.M.L.); caroline.lee@csiro.au (C.L.); 2 Agriculture and Food, Commonwealth Scientific and Industrial Research Organisation (CSIRO), Glen Osmond, SA 5064, Australia; jackie.ouzman@csiro.au (J.O.); damian.mowat@csiro.au (D.M.); rick.llewellyn@csiro.au (R.S.L.)

**Keywords:** behavior, GPS, LAI, time budget, lying time, regenerating, pasture

## Abstract

**Simple Summary:**

The eShepherd^®^ virtual fencing system being commercialized for cattle has the potential to exclude cattle from environmentally sensitive areas. Animals are given audio cues to indicate a fence line via a neckband device. An electrical pulse is administered if the animal continues moving forward following an audio cue. A commercial trial was conducted in South Australia to assess whether virtual fencing technology could exclude 20 cattle from an area of regenerating saplings, across 44 days, using a contoured fence line. The results demonstrated that the cattle were able to rapidly learn the virtual fencing cues, responding primarily to the audio cue alone, and were excluded from the regenerating area for 99.8% of the trial period. Behavioral time budgets measured by automated devices on the leg changed across the trial duration, but in no consistent pattern. At the trial conclusion, the feed available in the protected zone was double the quantity and quality of the grazed zone. Thus, virtual fencing technology using pre-commercial prototypes was shown to protect an environmental asset within a paddock from cattle grazing in the presence of a large feed differential.

**Abstract:**

The eShepherd^®^ virtual fencing system being commercialized for cattle has the potential to exclude cattle from environmentally sensitive areas. Animals are given audio cues to indicate a fence line via a neckband device. An electrical pulse is administered if the animal continues moving forward following an audio cue. A commercial trial was conducted in South Australia to assess whether virtual fencing technology could exclude 20 cattle from an area of regenerating saplings; across 44 days; using a contoured fence line. The results showed that the cattle were able to rapidly learn the virtual fencing cues; responding appropriately to the audio cue for 74.5% of 4378 audio signals; and were excluded from the regenerating area for 99.8% of the trial period with the more complex fence line (contoured; not straight) in place. IceQube R’s^®^ measuring lying time and bouts showed no consistent increasing or decreasing pattern of change. At the trial conclusion; the feed available in the protected zone was double the quantity and quality of the grazed zone. Technical issues occurred with some of the pre-commercial prototype devices; but those versions are now obsolete. This study observed a single group of cattle in one paddock; further testing of the virtual technology is warranted.

## 1. Introduction

Virtual fencing technology applies signals to control grazing livestock without using physical barriers. This technology has the potential to allow continuous animal monitoring, improve livestock management, reduce labor, and exclude animals from environmentally sensitive areas or otherwise challenging terrain. The eShepherd^®^ virtual fencing system being commercialized for cattle by Agersens (Melbourne, VIC, Australia) uses licensed IP (Intellectual Property) developed by the Commonwealth Scientific and Industrial Research Organisation (CSIRO) [1,2,3,4]. This system employs GPS technology, a base station, and individual neckbands worn by cattle with no buried wire components. Virtual fences are drawn via an online user-interface and their location is communicated to the animal using an audio tone as the animal approaches the boundary, followed by an electrical pulse if the animal continues moving forward. This signal pattern harnesses the associative learning capabilities of the animals so that they can avoid receiving an electrical pulse by learning to stop or turn away from the virtual boundary when the audio tone is emitted. Thus, the system is developed as ethical and welfare-friendly with controllable and predictable cues for the animals [5]. 

Several trials have been conducted within Australia using varying iterations of the eShepherd^®^ pre-commercial prototype neckbands on both beef and dairy cattle [6,7,8,9,10]. Those trials have been conducted on small groups of cattle (maximum 12 animals) in both experimental and commercial settings using straight virtual fence lines. The technology can exclude cattle from prescribed areas up to a tested period of 27 days [9] with individual cattle showing high variation in both how rapidly they learn to respond to the audio cue alone, and how often they continue to interact with the virtual boundary across the trial period. The technology has been demonstrated to keep individual beef cattle away from a rewarding food source [7], to periodically exclude beef cattle from different paddock sections with moving fence lines [6], to temporarily exclude beef cattle from a riparian zone [8], and to manage dairy cattle in different grazing allocations [10]. Research has also demonstrated that across a four-week period, virtual fencing was comparable to electric tape fencing in its impacts on the behavioral time budgets of small groups of grazing beef cattle, their fecal cortisol metabolite concentrations, and paddock utilization patterns [9]. 

Management of cattle around environmentally sensitive areas is a key potential benefit of virtual fencing systems [11]. Whether this is through using temporary fences that can exclude cattle for specific periods of time to allow grazing control of natural areas leading to increased biodiversity, and/or exclusion from areas that may be challenging to physically fence such as waterways or small portions of grazing paddocks [12,13,14,15,16]. A previous trial demonstrated the ability of manually operated virtual fencing collars to exclude sheep from an area of potentially erodible soils in a small paddock over 3 days [17]. Earlier pre-commercial prototypes of the automated neckbands used in the current study kept a group of 11 cattle away from a river area on a commercial property for 10 days, with the cattle accessing the area again within a few hours of the virtual fence being removed [8]. The potential environmental management scenarios for virtual fencing warrant further testing to measure the applications and limitations of the technology. 

The objectives of the current study were to utilize the pre-commercial eShepherd^®^ system to exclude a larger group of cattle from a native vegetation regeneration area on a commercial property for an extended period of time, using a contoured fence line. Previous virtual fencing trials have relied on single straight-line fencing and have not tested learning and efficacy with contoured fence lines, which are likely to be necessary in many environmental protection applications. When protecting an environmental asset, the likely incursion distance and necessary setback distance of the virtual fence line from the asset also become important considerations. These were investigated in the study along with measures of individual animal learning, behavioral time budgets, and pasture composition. It was predicted that the technology would limit animal access into the excluded area, resulting in a pasture differential, and that the cattle would be capable of learning a more complex fence line but with high individual variation. Normal behavioral time budgets were anticipated. 

## 2. Materials and Methods 

### 2.1. Ethical Statement

The experiment was approved by the CSIRO FD McMaster Laboratory Chiswick Animal Ethics Committee (ARA19/02) before the start of the experimental period.

### 2.2. eShepherd^®^ Neckbands

The virtual fencing pre-commercial prototype (eShepherd^®^, Agersens, Melbourne, VIC, Australia) system was used in these trials and has been described previously [8,9]. The specific version of the pre-commercial prototype used has since been rendered obsolete following design refinements. The neckband worn by the cattle consisted of a strap and hanging counterweight (total weight approximately 1.4 kg) and a unit (approximately 725 g and 17 cm L × 12 cm W × 14 cm H), positioned on the top of the animal’s neck. Using GPS technology, the unit monitored the animal’s movement to provide a real-time measure of the animal’s position, heading, and speed. A virtual fence boundary (separating inclusion versus exclusion zones), was specified using GPS coordinates, and transmitted to the unit using a radio frequency link. As an animal approached the virtual fence boundary, the unit emitted a distinctive but non-aversive audio tone within the animal’s hearing range. If the animal stood still or turned away, no electrical pulse was applied. If the animal continued to move through the virtual fence boundary into the exclusion zone, the unit delivered a short, sharp electrical pulse sequence in the kilovolt range. The intensity of the pulse stimulus delivered by the neckband was lower in energy than an electric fence. Additionally, because the neckband was worn by the animal, the pulse was delivered via a different mechanism than an electric fence. Hence no direct comparison can be made of pulse intensity between a neckband and a standard electric fence. The precise values of the electrical pulse are commercial-in-confidence. This sequence of an audio cue followed by the electrical pulse was repeated if the animal walked through the fence line and continued into the exclusion zone. No stimuli were applied if the animal turned around to re-enter the inclusion zone. This algorithm design functions to ‘herd’ the animals back out of the exclusion zone after entry. If animal movement occurred above or below a specified velocity (values are commercial-in-confidence), stimuli were not applied. As a safety feature to limit the maximum number of consecutive pulses an animal could receive, the device entered standby mode and stimuli were not applied for a specified time frame (values are commercial-in-confidence) if an individual received a specified number of pulses within a specified timeframe (values are commercial-in-confidence). The neckband algorithm also included a ‘grazing function’. The natural behavioral pattern of grazing can mimic the correct response by the animal to the neckband cues of movement forward and stopping at an audio cue. Therefore, if an animal received 3 consecutive audio cues while still moving forward paired with stopping, an electrical pulse was applied. A base station was set up adjacent to the trial paddock that communicated with the neckbands, and animal activity was able to be monitored in real time through an online user-interface. All neckband cues were stored on a removable SD card within the device for later download. 

### 2.3. Animals, Site, and Experimental Protocol

**Figure 1:**
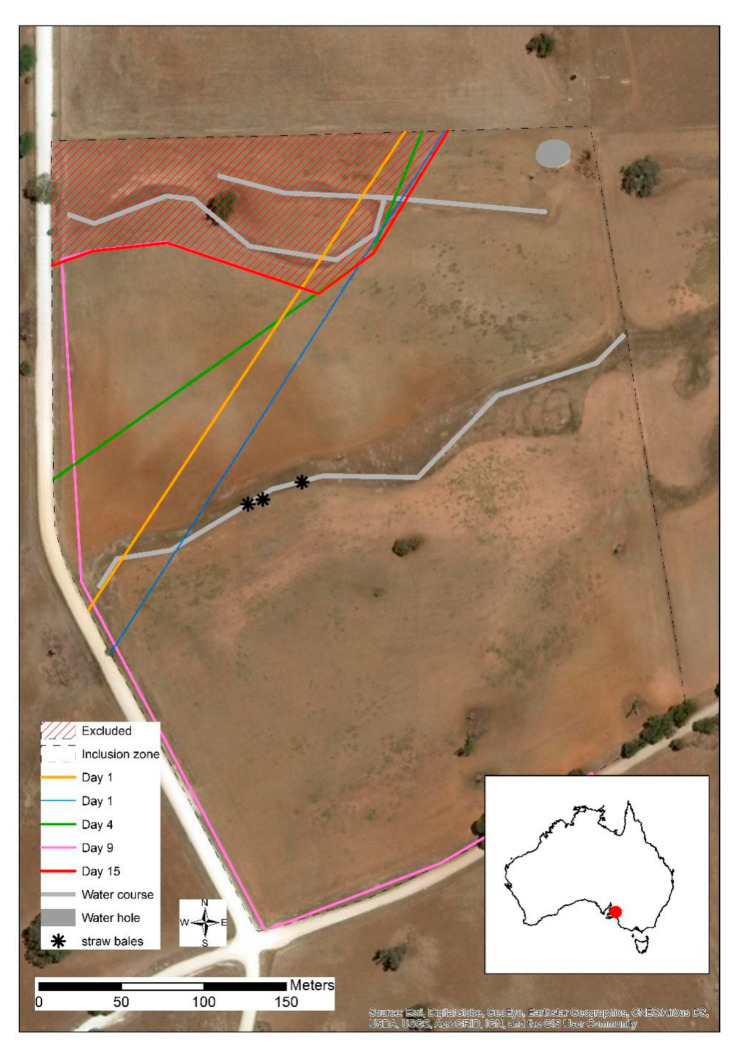
The commercial paddock, regenerating sapling area (excluded area), cattle drinking water, water courses (that were empty at the time of the trial) and the progression of virtual fences that were set throughout the days of the trial as indicated. Placement of supplemental straw bales are indicated.

The trial was conducted on a 14-ha wire-fenced paddock (Figure 1) within a 770-ha commercial grazing property in Eden Valley, SA from May until July 2019 (autumn/winter season). The paddock was primarily Lucerne (*Medicago sativa*) pasture with an area of regenerating Eucalyptus (*Eucalyptus globulus*) saplings in the upper section of the paddock (Figure 1). The saplings were up to approximately 5 years old and ranged in height from 0.5 m to 2.5 m. Dry watercourses were comprised of mixed grass growth. One water point within the paddock was available to cattle ad libitum (Figure 1). Animals were given one supplementary barley straw bale weighing approximately 300 kg DM, on days 10, 16, 32, and 36 (see Figure 1 for bale placement). On day -2, 20 Santa Gertrudis heifers (10–12 months old, mean body weight ± SE 369.85 ± 7.02 kg as weighed by Gallagher Smart TSi and weigh bars, Gallagher Australia Pty, Ltd., Epping, VIC, Australia), with no prior exposure to virtual fencing, were fitted with the eShepherd^®^ pre-commercial neckband prototypes and an IceQube R^®^ (IceRobotics Ltd., Edinburgh, Scotland, UK) on the front leg of each animal to measure daily standing, lying, and lying bouts. The animals were kept in an adjacent paddock to the test paddock for 3 days to allow some acclimation to the devices and to fit with farm and research personnel schedules. On day 1, all cattle were moved to the test paddock and a straight virtual fence training line was immediately set to exclude cattle from the sapling area (Figure 1). The first virtual line deliberately exposed some of the saplings to enable a comparison between protected and exposed saplings. However, this fence line was shifted approximately 5 m in a SE direction within two hours to include all saplings in the exclusion area (Figure 1), once it was apparent that the saplings could be rapidly destroyed by the cattle (observations from a distance showed that the animals were knocking against the trees, rubbing and mouthing them). All animals were observed by personnel located in the adjacent paddock for their first interactions with the virtual fence line across the daylight period of the first day. Those observations were to ensure that there were no extreme behavioral reactions that may lead to injury or distress (e.g., running into physical fences, circling, continual vocalizations) and that the animals were learning to turn away from the virtual fence. Previous trials indicated that animals start appropriately responding to the virtual fence within the first day [9]. On day 4, the fence line was shifted to the next angled training iteration (Figure 1). On day 9, the fence line was redrawn to include a virtual barrier against part of the physical fence (Figure 1) to prevent the cattle from pushing their heads through the physical wire fence and removing the neckbands. The neckbands were likely removed via the button electrodes getting caught on the wire (the most plausible explanation based on opportunistic observations, *n* = 3 occurrences). The refined design of the pre-commercial prototypes no longer includes button electrodes. However, to conserve battery power on the devices, on day 15, electrical wire was placed along the physical fence on the western and southern sides of the paddock and the virtual fence line was redrawn as contoured along the sapling area (Figure 1). This final virtual fence remained in place until the conclusion of the trial on day 44. On day 12, two animals with battery malfunctions, and, on day 14, one animal whose neckband came off, were removed from the paddock. On the afternoon of day 18, all animals were moved to adjacent yards for approximately 24 h and refitted with an updated pre-commercial prototype neckband that improved upon previous firmware issues associated with battery charging. As the devices were pre-commercial prototypes, the improvements being made within the firmware and software were continual. Updated devices were applied to reduce battery-charging issues for the remainder of the trial. All 20 animals were returned to the experimental paddock. On day 36, three animals started pushing through into the exclusion zone; on day 39, those three animals (Q26, Q29, Q36) were removed from the paddock. On day 44, the trial concluded; all animals (*n* = 20) were weighed (mean ± SE: 354 ± 7.44 kg) and all devices removed.

### 2.4. Pasture Assessments

**Figure 2:**
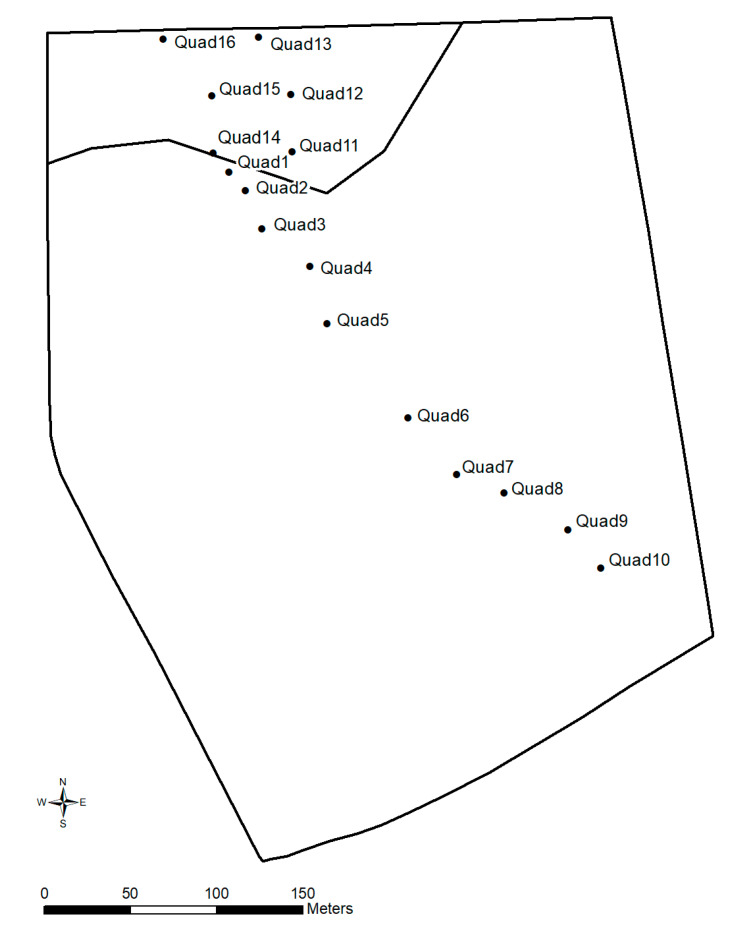
Location of quadrat cuts for pasture assessments across the paddock. Quadrats 1–10 were sampled four days before the start of the trial, quadrats 1–16 were sampled two days after the conclusion of the trial. The final contoured virtual fence line separating the smaller exclusion and larger inclusion zone is indicated.

Four days before the start of the trial, an assessment of pasture availability and quality was performed using a transect of ten 2 m × 2 m pasture quadrat cuts across the paddock (Figure 2). The quadrat locations were selected along a transect across the paddock with the sampled area selected as representative of that part of the transect. This was repeated at the end of the trial, two days after the 44-day grazing period with 16 pasture cuts sampled (inclusion zone *n* = 10, exclusion zone *n* = 6, Figure 2). Nutritional value of the pasture sub-samples was determined at the CSIRO laboratory in Floreat, WA, using near infrared spectroscopy (NIRS) calibrated with wet chemistry. The NIRS was a Unity SpectraStar 2500x rotating top window system (Unity Scientific, Emu Plains, NSW, Australia). Detailed descriptions of the NIRS calibration development, accuracy statistics and wet chemistry methods, are presented in [18]. Total nitrogen was determined by combustion using a Leco CN628 N Analyser [19] and crude protein (CP) was calculated by total N × 6.25. In vitro dry matter digestibility (DMD) was determined in duplicate using a modified pepsin-cellulase technique as previously described [18,20]. 

Leaf Area Index (LAI) measurements and NDVI-based (Normalized Difference Vegetation Index) mapping of the paddock were also conducted on day 46 across the whole paddock using a Crop Circle ACS-430 and Crop Circle Phenom (ACS-430 with DAS43X, Holland Scientific, Lincoln, NE, USA) respectively. These measurements were used to generate a kriged map of pasture across the paddock showing the impact of the grazing exclusion on biomass and groundcover-related measures. LAI and NDVI were highly correlated, so only an LAI map (generated using ArcMap as per the protocol of [21]) is presented. 

### 2.5. Data and Statistical Analyses

All neckband data per animal (*n* = 20) for each trial day (*n* = 44) were collated to obtain GPS locations, animal distance relative to the virtual fence line and delivered audio and electrical stimuli. A total of 14,499,072 GPS records were obtained for the duration of the trial. GPS data were recorded at approximately one second intervals when the neckbands were ‘active’ (i.e., if an animal was close to the fence line) but this frequency reduced when the animals were distant from the fence line (exact specifics of the algorithm are commercial-in-confidence). For the 20 animals, over 44 days, we collected 833 records out of a potential 880 daily records, with missing records attributable to the animal management factors described in the methods and some occasional technical malfunction in data storage (between 36 and 44 records/animal). GPS data were plotted using the point density tool in ArcMap within the ArcGIS software suite (v. 10.41, Esri, Redlands, CA, USA). Grid cells (2 m × 2 m) were selected across the paddock area where the tool then counted the GPS point in and around each cell. The density of GPS points in each grid was divided by the number of days for a specific map to provide an average per day pattern of paddock occupancy for the group (e.g., total GPS counts divided by 3 days for the first day’s 1 to 3 map). 

The neckband data were also analyzed to summarize each time an animal entered the exclusion zone (i.e., when they crossed over the virtual ‘line’: termed one event) for each day of the trial, including the total time that individual animals spent in the exclusion zone across the trial duration (at different distances into the exclusion zone), the maximum distance into the exclusion zone they travelled per event, and the total time spent past the virtual line across the trial duration. This enabled calculation of the summed total duration of time that animals spent in the exclusion zone across the 998 h of trial duration. The audio and electrical pulse cue data were summarized to show the total number of audio and pulse cues received per individual animal across the trial duration and an overall proportion of audio-only (i.e., audio cues not followed by an electrical pulse) for all animals across the trial duration (excluding the 3 animals from when they began entering into the exclusion zone at day 36 onwards as a likely result of device unreliability around pulse delivery).

All daily behavioral data from the IceQube^®^ R’s were compiled per individual animal with data missing from 3 animals due to device malfunction (reason for malfunction was unknown). Data were not included from days 18, 19, and 43 as these were incomplete days of data. Data from animals that were temporarily removed from the test paddock (as indicated in Section 2.3) were not included for those days spent outside the test paddock. The lying time and number of lying bouts data were plotted with the maximum daily temperature and total daily rainfall across the trial period as obtained from weather stations located approximately 36 km and 10 km away from the trial site, respectively, (http://www.bom.gov.au/climate/data/). It must be acknowledged that the individual animals were located in a single paddock, were not independent from each other and there was no control comparison group, thus limiting statistical analyses.

The values for DMD and CP were not normally distributed and were thus analyzed using non-parametric Wilcoxon signed-rank tests to compare values at the end of the trial between the inclusion and exclusion zones. Analyses were conducted in JMP 14.0 (SAS institute, Cary, NC, USA) with α set at 0.05.

## 3. Results

### 3.1. Animals

**Figure 3:**
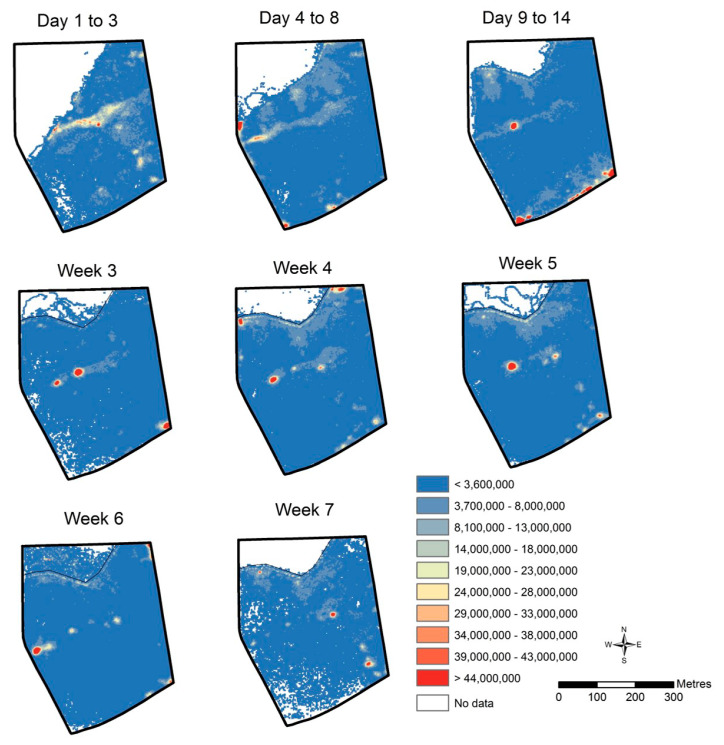
The per day GPS plots of animal occupancy within the test paddock across time, with progressive iterations of the virtual fence line. GPS data were recorded at approximately one second intervals when the neckbands were ‘active’ (i.e., if an animal was close to the fence line), but this frequency reduced when the animals were distant from the fence line. The GPS data were then plotted using the point density tool in ArcMap 10.3 using grid cells (2 m × 2 m) across the paddock area. The frequency of GPS points of each grid was divided by the number of days for a specific map to provide the average per day pattern. Three animals were removed in week 7 after their devices were malfunctioning during week 6, resulting in more time spent in the exclusion zone (see Methods and Results text for more details).

**Figure 4:**
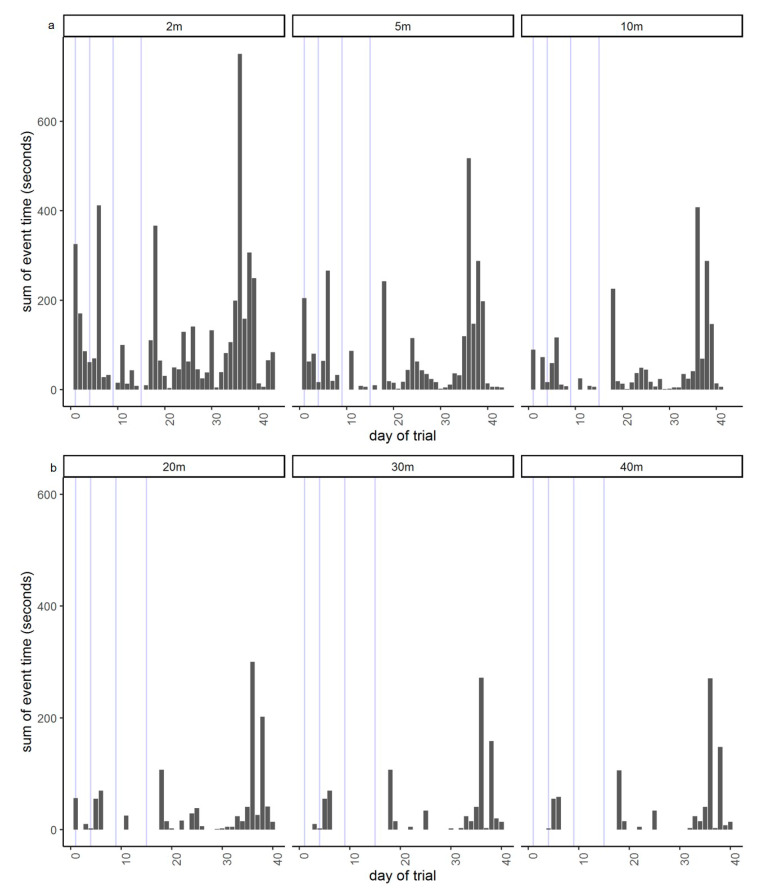
The sum time spent in the exclusion zone at different minimum distances ((**a**): 2, 5, 10 m; (**b**): 20, 30, 40 m) from the virtual fence line across all animals for the 44-day trial period. The first plot of 2 m includes all incursion events with the remaining plots indicating which portion of these incursions had the respective minimum distance travelled (e.g., at 5 m, these animals went at least 5 m into the exclusion zone). The blue lines indicate the activation of different virtual fence lines on days 1, 4, 9, and 15, respectively (as per Figure 1). Animals Q26, Q29, and Q36 were removed on day 39 of the trial as they were regularly crossing into the exclusion zone at greater distances from days 36 to 39, which was later attributed to their devices recording irregularly low pulse current values.

**Figure 5:**
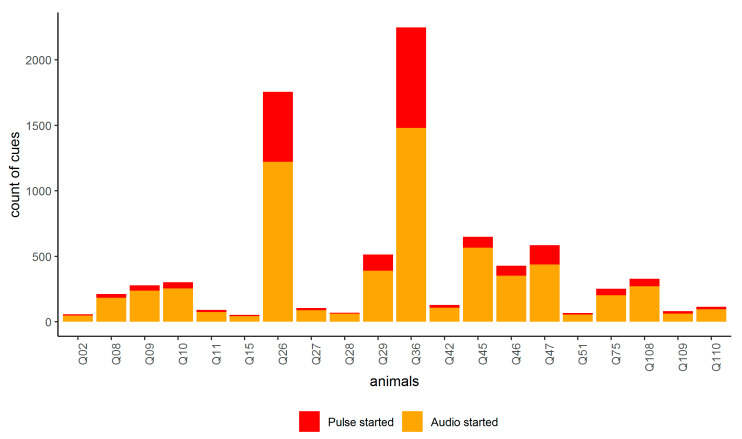
The number of audio and electrical pulse cues that individual animals received across the 44-day trial. Animals Q26, Q29, and Q36 were removed on day 39 of the trial as they were regularly crossing into the exclusion zone at greater distances from days 36 to 39, which was later attributed to their devices recording irregularly low pulse current values.

**Figure 6:**
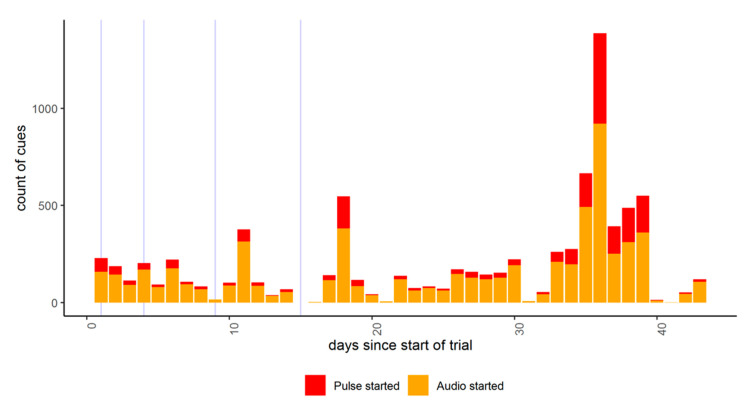
The number of audio and electrical pulse cues that all animals received across the 44-day trial. The blue lines indicate the activation of different virtual fence lines on days 1, 4, 9, and 15, respectively (as per Figure 1). Three animals were removed on day 39 of the trial as they were regularly crossing into the exclusion zone from days 36 to 39, which was later attributed to their devices recording irregularly low pulse current values.

GPS plots showed that animals were contained within the inclusion zone and utilized all areas of the available paddock. Of the total 998 h within the trial, a summed total of 1 h 47 min across all animals were spent past the virtual boundary; thus, 99.8% of time, the animals were excluded (Figure 3 and Figure 4a). At least one animal did cross into the exclusion zone on every day of the trial except for two (days 9 and 15), with a mean daily number of incursion events greater than 2 m across all animals of 2.7 ± 3.5 SD (interquartile range: 2; data range: 1 to 33). There was a total of 756 incursion events ranging from 0.002 m to 77 m (the depth of the exclusion zone) with 403 of those greater than 2 m. Within those 403 incursions, 41 were greater than 40 m past the virtual line and 27 of these were attributed to the three animals that were eventually removed (Figure 4a,b). The mean time spent in the exclusion zone during the incursion events across all animals was 5 min 36 s (interquartile range: 3 min; range: 31 s to 17 min 27 s). Similarly, the increased incursions, as seen in week 6 in Figure 3, were also mainly due to the three animals (Q26, Q29, and Q36) who were subsequently removed on day 39 of the trial due to malfunctions in pulse strength delivery. Of the total 403 incursions past 2 m within the entire trial, the three removed animals completed 160 of those. Other animals (Q45, Q46, Q47) showed similar numbers of cues to Q29 (Figure 5), but they were not crossing into the exclusion zone as frequently (56 total incursions together across the trial duration) and thus were left in the trial. The number of audio and electrical pulse cues that animals received varied across days of the trial with a greater increase towards the end of the trial that was related to the animals that were starting to regularly cross over into the exclusion zone before they were removed (Figure 5 and Figure 6). There was also high individual variation with the most cues received by two of the animals that were removed (Figure 5). Analysis of the strength of the delivered pulse current showed that animals Q26, Q29, and Q36 had double the standard deviation in values relative to the other cattle in the 4 days before their removal from the trial (the specific pulse current values were available to the researchers for analysis but are unable to be presented due to being commercial-in-confidence). This indicated that the pulse current was lower than expected during this period. All animals received a higher number of audio cues than electrical pulse cues, indicating they were learning the cue association (Figure 5) with 74.5% of a total of 4378 audio cues being audio-only (i.e., not followed by an electrical pulse: audio to pulse ratio was 0.8). 

**Figure 7:**
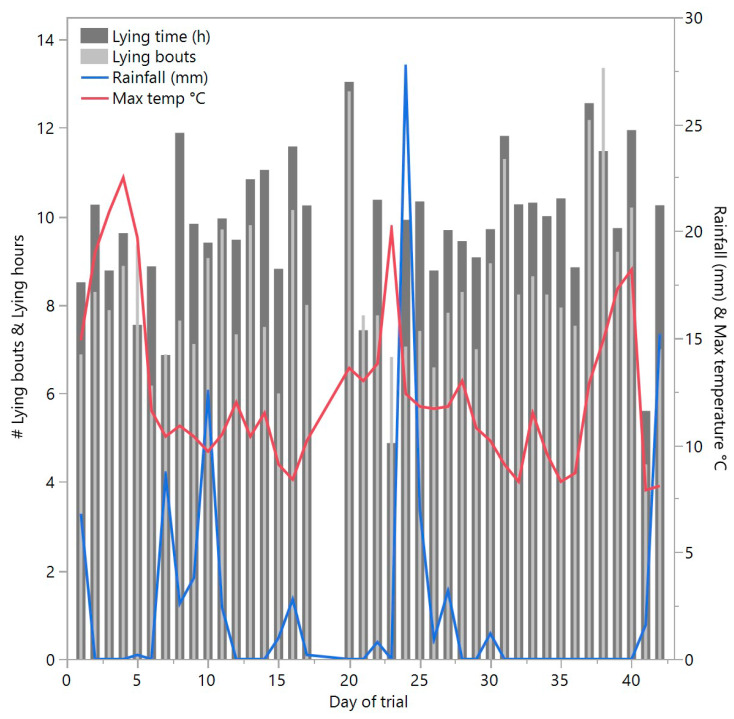
The raw data of mean lying time (hours) and number of lying bouts for cattle on each day of the trial (excluding incomplete data on days 18, 19, and 43), including maximum daily temperature and daily rainfall as measured by weather stations approximately 36 km and 10 km away, respectively.

Descriptively, there was high day-to-day variation in both lying time and lying bouts but in no consistent pattern across time (Figure 7, mean lying time ± SD 9.76 ± 1.93, mean lying bouts ± SD 8.38 ± 2.81). There were no visibly consistent associations with rainfall and maximum temperature records (Figure 7). 

### 3.2. Pasture 

**Figure 8:**
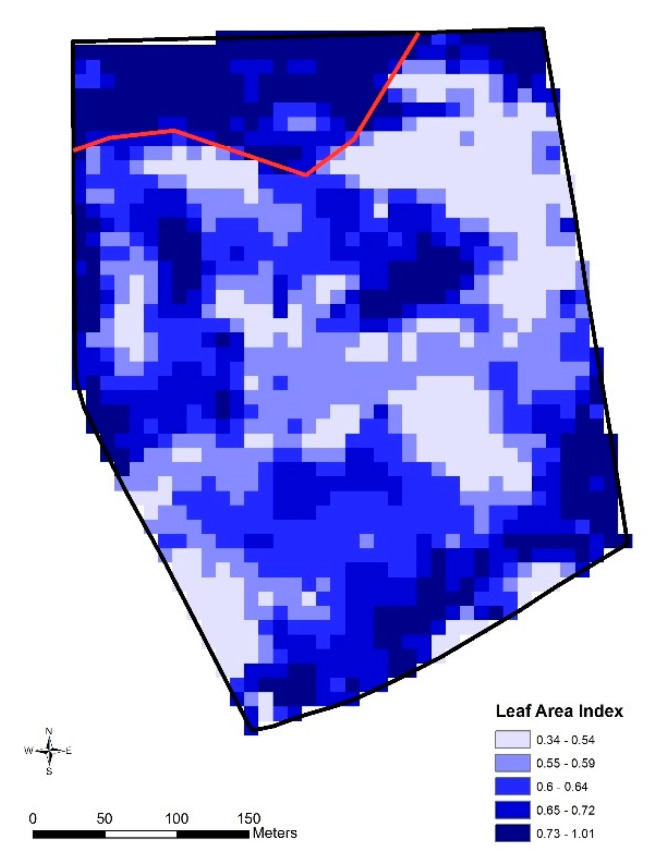
Leaf Area Index showing the virtual fence line and higher pasture vegetation within the exclusion zone at the conclusion of the trial. The saplings within the exclusion zone did not contribute to these measurements.

At the beginning of the trial, before substantial early-season regrowth had occurred, the grazing zone contained an average biomass of 0.50 t/ha with an average DMD of only 35.4% (SE ± 1.1%) and crude protein of 6.8% (SE ± 0.5%). This reflected the dominance of dry pasture residue and only low levels of emerging fresh regrowth at that time. At the conclusion of the trial, the biomass in the grazed inclusion zone was 0.31 t/ha (SE 0.07) and 0.59 t/ha (SE 0.15) in the non-grazed exclusion zone, based on the average of the quadrat cuts in each zone (Figure 2). The exclusion zone had significantly higher DMD and CP (both *Z* = 3.2, *p* = 0.0014) than the inclusion zone (mean ± SE DMD inclusion zone: 31.6 ± 1.1%, exclusion zone: 53.6 ± 3.8%; CP inclusion zone: 6.1 ± 0.7%, exclusion zone: 20.7 ± 2.1%). The substantial differential in pasture biomass and groundcover was reflected spatially in the post-trial LAI map of the paddock, which showed consistently high vegetation cover within the exclusion zone compared to large areas with low LAI in the grazed zone (Figure 8). There was no damage observed in the saplings at the trial conclusion. 

## 4. Discussion

This trial aimed to assess whether virtual fencing technology could exclude a group of 20 cattle from an environmentally sensitive area containing regenerating saplings, across a period of 44 days, on a commercial property, using a contoured fence line. The results demonstrated that the cattle were able to rapidly learn the virtual fencing cues, respond to the audio cue alone, and were excluded from the regenerating area for 99.8% of the (total animal hour) trial period with the more complex fence line in place. While behavioral time budgets of lying time and bouts changed across the trial duration, they were in no consistent increasing or decreasing pattern. At the end of the trial, the feed available in the protected zone was approximately double the quantity and quality of the inclusion/grazed zone. Virtual fencing technology was shown to protect an environmental asset within a paddock from cattle grazing in the presence of a large feed differential across the fence line, but the study was conducted on only one group of animals in a single paddock, and thus replication with updated pre-commercial prototype devices is needed.

Similar to multiple past applications of the automated eShepherd^®^ system with beef cattle [6,7,8,9], all animals showed the ability to associatively learn and respond to the audio cue alone, in order to avoid receiving electrical pulses, but with high individual variation in the rate at which they learnt, and also the frequency at which they continued to interact with the fence line. The animals approached the fence throughout the trial period, indicating that they were not actively avoiding that area. The ratio of audio to pulse cues showed that the animals did not receive comparatively excessive pulse stimuli around the contoured virtual boundary compared with what has been observed with straight fence lines [9]. This confirms the ability of cattle to be attuned to the audio cue and modify their movement behavior accordingly, to stay within the inclusion area [6]. Animals did cross into the exclusion zone, but fewer instances of travel farther into the zone were recorded compared with movement a few meters past the virtual line (see Figure 4a,b). Extended periods of time, up to approximately 20 min, were observed before animals were herded back into the inclusion area by the virtual fencing technology (animals continued to receive both audio and pulse signals as they ventured farther into the exclusion zone, encouraging them to turn around and walk back out). The pasture differential at the end of the trial indicated that the technology was able to exclude animals under high grazing pressure. However, this feed differential may have contributed to the motivation of some individuals to enter the exclusion zone, particularly as animals were losing weight across the trial duration with the low available paddock feed (and some straw supplementation). Although there was no observable damage to the saplings resulting from the time the animals spent in the excluded area, it is highlighted that the virtual fence may not provide 100% animal exclusion (similar to standard electric fences), and that it would be recommended to set the fence some distance in front of the area needing to be protected, to create a buffer zone around sensitive areas.

Toward the end of the trial, there were a few individual cattle that were repeatedly breaking through the virtual fence line and entering the exclusion zone. The three individuals that were removed received substantially higher numbers of audio and electrical cues relative to the rest of the group and previous studies [9]. The pulse current values in these animals indicated that the pulse current was low and variable and was not sufficiently aversive; the animals were ignoring both it and the audio. The likely cause was a prototype reliability issue in these earlier prototypes, for which the device design has since been modified. Although the incursion by the animals with those neckbands did not result in the rest of the herd following them in, the animals were removed to minimize this risk. Studies have shown that individual cattle can be unresponsive to standard electric fences, with the need to remove those animals [22]. However, despite the lack of compliance late in the current trial, the three animals were successfully excluded by the virtual fence for most of the study, so they were initially responsive to learning the technology. While the devices used in the current study functioned for the majority of the trial, there were several technical issues that required animals to be temporarily removed and devices replaced following updates. The level of monitoring and intervention required in this research trial would not be practical in a commercial setting, but it must be acknowledged that the version of the prototype device used is now obsolete and improved functionality of refined prototypes is the subject of current testing and research.

The behavioral time budgets of lying time and lying bouts showed daily changes across the trial duration, but in no consistent pattern, and thus were likely related to natural expected variation across time. The mean daily lying time for the animals across the trial showed high fluctuations between days, with low hours on some days (approximately 5 to 6 h) that were typically followed by an increase the following day. The overall behavioral time budget results are comparable to reports of average lying time in both beef and dairy cattle across multiple studies [23,24,25]. Other studies have also reported variation across study days [26] and between individual animals [23,26], and low lying hours e.g., 5.1 h/day [27]. Thus, this variation was likely due to natural patterns which may have been partly associated with weather parameters. Further reports of how cattle on pasture vary in lying time across days is warranted. There was only a single group of cattle assessed in this study and no control group for comparison, but a recent study comparing several groups of cattle exposed to virtual fences or electric tape fences found a difference of less than 20 min/day in lying time between those treatment groups [9]. Future applications of the virtual fencing technology over several months, and including control groups, would further validate the impacts of such systems on typical cattle behavior to ensure the system is welfare friendly.

## 5. Conclusions

In conclusion, the virtual fencing technology was successful at excluding cattle from an environmentally sensitive area across a period of 44 days, on a commercial property, using a progressive introduction of a contoured fence line. Further work would replicate with additional groups of animals in different paddocks, and continue to test the complexity of fence lines that cattle can respond appropriately to. An extended trial period would further assess the factors that may lead to animals regularly crossing into the exclusion area. Use of updated pre-commercial prototypes would confirm functionality of the refined designs.
